# Exploring the association of Brownfield remediation status with socioeconomic conditions in Wayne County, MI

**DOI:** 10.1007/s11356-023-26666-2

**Published:** 2023-04-11

**Authors:** Brendan F. O’Leary, Alex B. Hill, Colleen Linn, Mei Lu, Carol J. Miller, Andrew Newman, F. Gianluca Sperone, Qiong Zhang

**Affiliations:** 1 Department of Civil and Environmental Engineering, Wayne State University, 5050 Anthony Wayne Dr., Detroit, MI 48202, USA; 2 Department of Urban Studies and Planning, Wayne State University, Detroit, MI, USA; 3 Department of Anthropology, Wayne State University, Detroit, MI, USA; 4 Department of Public Health Science, Henry Ford Health, Detroit, MI, USA; 5 Department of Environmental Science and Geology, Wayne State University, Detroit, MI, USA

**Keywords:** Brownfields, Urban, Environmental mapping, Environmental justice, Leaking underground storage tank

## Abstract

Urban neighborhoods with locations of environmental contamination, known as brownfields, impact entire neighborhoods, but corrective environmental remedial action on brownfields is often tracked on an individual property basis, neglecting the larger neighborhood-level impact. This study addresses this impact by examining spatial differences between brown-fields with unmitigated environmental concerns (open site) and sites that are considered fully mitigated or closed in urban neighborhoods (closed site) on the US census tract scale in Wayne County, MI. Michigan’s Department of Environment, Great Lakes, and Energy’s leaking underground storage tank (LUST) database provided brownfield information for Wayne County. Local indicators of spatial association (LISA) produced maps of spatial clustering and outliers. A McNemar’s test demonstrated significant discordances in LISA categories between LUST open and closed sites (*p* < 0.001). Geographically weighted regressions (GWR) evaluated the association between open and closed site spatial density (open-closed) with socioeconomic variables (population density, proportion of White or Black residents, proportion of college educated populations, the percentage of owner-occupied units, vacant units, rented units, and median household value). Final multivariate GWR showed that population density, being Black, college education, vacant units, and renter occupied units were significantly associated (*p* < 0.05) with open-closed, and that those associations varied across Wayne County. Increases in Black population was associated with increased open-closed. Increases in vacant units, renter-occupied units, and college education were associated with decreased open-closed. These results provide input for environmental justice research to identify inequalities and discover the distribution of environmental hazards among urban neighborhoods.

## Introduction

Urban communities in the USA that came “of age” during the Industrial Revolution endure a legacy of toxic contaminants and hazardous waste, with neighborhoods of color and lower income often bearing the greatest impacts (Collins et al. 2016; Eckerd and Keeler 2012; Filippelli et al. 2015; Litt et al. 2002). The sites of environmental contamination, referred to as brownfields, are complicated by the presence of a hazardous substance, pollutant, or contaminant (Office of Brownfields and Land Revitalization 2022). Brownfields are a source of health risks and property devaluation for those who live nearby (Brender et al. 2011; Gomez et al. 2017; Lee and Mohai 2011; Litt et al. 2002). Frequently, brownfields are prioritized for remediation based on land development goals, potential pro-economic opportunities, community interest, exposure risk, available resources, and other extenuating variables (Brownfield Redevelopment Program 2021; Lee and Mohai 2012; Reddy and Adams 2015). Given the historical association of brownfield investment with land development, for this study, we hypothesized that socioeconomic variables commonly connected with increased affluence (e.g., property ownership, wealth, and education outcomes) are associated with brownfield investment.

The state of MI is recognized as having one of the most innovative programs addressing brownfield remediation (Hula and Davis 2004; Meerow et al. 2019). However, there is a lack of data on overall program efficacy. Most often, researchers focus on a single site or case study (Leigh and Coffin 2005), failing to capture a broad view of the program features. In addition, owners do not want to bring attention to specific properties being remediated, and, as a result, communication of risk between parties is often low (Wu et al. 2017). Economic interests can overcome significant barriers to remediation or redevelopment by providing initial cleanup funds and long-term funding, working with market conditions, and reducing owner liability (Goodstein et al. 2011; Hula and Bromley-Trujillo 2010). When remediation occurs, the appropriate cleanup level is determined based on site reuse criteria (e.g., residential, commercial, industrial). Often, no remediation is physically performed; instead, a containment approach is employed leaving legacy contaminants in place (Brownfield Redevelopment Program 2021; Smith 2019). Such sites receive closure status (“closed”), although the contamination remains.

This study targets leaking underground storage tank (LUST) locations in Wayne County, MI, and evaluates the differences between LUST open sites and LUST closed sites. The terminology of “open” and “closed” arises from Michigan Law Part 213 (Leaking Underground Storage Tanks) of the Natural Resources and Environmental Protection Act, Public Act 451 of 1994, as amended (Act 451). In the USA, LUST sites are regulated by state and local authorities with minimum standards set by the US Environmental Protection Agency. An open LUST site is one requiring additional corrective action. A closed LUST site has achieved a regulatory endpoint. This endpoint is generally achieved through either active remediation or, more often, engineering controls (e.g., land use restrictions) that mitigate environmental risk exposure (Favara et al. 2019; Office of Brownfields and Land Revitalization 2022). Consequently, many closed sites, while meeting regulatory standards to mitigate environmental risk, often contain environmental toxins and present environmental justice concerns (Currie et al. 2015; Wilson et al. 2017; Wilson et al. 2013). Closed sites represent a favorable status when compared against open sites. However, previous work has noted that improperly built protections, infrequent monitoring, and difficult enforcement of deed restrictions can occur (Greenberg 2002; Solitare and Greenberg 2002). Evaluating differences in the environmental remediation status over a region can provide insight into the high-level decision-making process regarding corrective action.

Wayne County, the focus of the present investigation, encompasses the city of Detroit, a majority-minority city, and inner-ring suburbs that vary widely in racial and ethnic compositions. The population density in Detroit significantly exceeds that in the surrounding suburban areas of Wayne County (1865 people/km^2^ in Detroit versus 1104 people/km^2^ in Wayne County) (US Census Bureau 2020). Considering both LUST open sites and closed sites in Wayne County, (1) we sought to identify brownfield distribution, (2) evaluate differences in spatial patterns of environmental remediation, and (3) evaluate these spatial patterns against local socioeconomic and property development factors. Brownfield sites associated with high property development potential and land value have historically been targeted for brown-field cleanup (Carozzi 2020; Chen et al. 2019; Haninger et al. 2017; Woo and Lee 2016). Therefore, we expected that closed sites may be associated with increased property ownership, wealth, and education outcomes compared to open sites.

## Methods

**Fig. 1:**
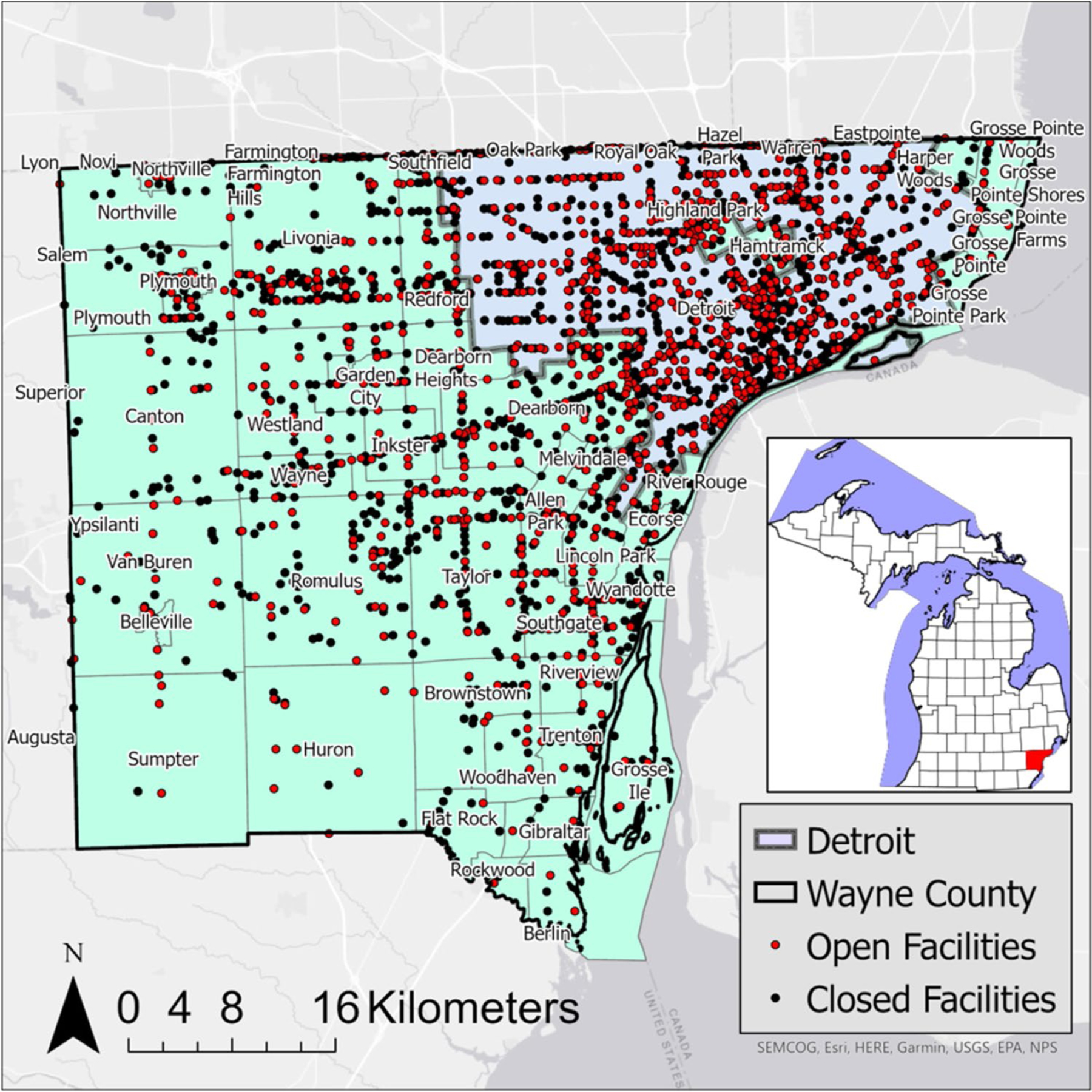
Spatial distribution of open and closed LUST facilities in Wayne County. The inset map shows the location of Wayne County within the state of MI

A variety of resources was used to document the 1236 open and 2072 closed LUST locations in Wayne County for this investigation. These include the Michigan Department of Environment, Great Lakes, and Energy (EGLE) Enviromapper (O’Leary 2020), EGLE’s SID database, and the LUST Legacy Site list. The LUST facilities are distributed over 59 municipalities in Wayne County (Fig. 1) and 610 US census tracts (US Census Bureau 2020). The LUST datasets collected through March 2021 and socioeconomic data were captured based on 2020 US Census Bureau American Community Survey. All LUST sites in the study were overseen by the same regional office at Remediation and Redevelopment Division within EGLE.

Geospatial analyses were the primary method used in the present investigation to understand the distribution of open and closed LUST sites with various demographic variables. Geospatial analyses that pair the demographic variables with closed/open status provide important insights into the potential decision variables involved in the likelihood of a site’s status as “closed” or “open.” Data were collected at the census tract level and normalized to area (km^2^).

The local indicators of spatial association (LISA) local Moran’s I statistic (Anselin 1995) examined the spatial clustering of both high and low densities of LUST sites as well as spatial outliers. For each location, LISA calculates the statistical significance and measures the local neighborhood (inverse distance) against the global dataset. Through the proportional relationship between the local and global statistics, LISA categorizes each location into high-value clustering (high-high: H–H), low-value clustering (low-low: L-L), high-value outliers (low–high: L–H), low-value outliers (high-low: H–L), or not statistically significant (N.S.). The McNemar’s chi-squared test (Pembury Smith and Ruxton 2020) was used to test agreement on all categories (H–H, L-L, H–L, L–H, or N.S.) between open sites and closed sites, and the statistical significance of the difference.

The association of open sites with local social and economic factors and property development variables was studied using the difference between open and closed site spatial density, called open-closed, as the outcome of interest and eight socioeconomic variables as the covariates. These covariates include population density, proportion of White and Black residents, and proportion of college-educated populations, as well as the following property variables: percentage of owner-occupied units, vacant units, rented units, and median household value (US Census Bureau 2020). Geographically weighted regression (GWR) in R (R Core Team 2013) was performed to estimate the bandwidth and its associated Akaike information criterion correction (AICc) via forward stepwise regression approach. The correlation between variables was assessed. To avoid collinearity, highly correlated variables, determined as correlation coefficient > 0.70, were included in the model one at a time along with other covariates. The final multivariable model was selected if it retained covariates with *p*-values < 0.05 and the model minimized AICc, called the optimal spatial search bandwidth and defined as the number of census tracts in the local neighborhood. The final model was further processed using ESRI ArcGIS Pro 2.7.0 GWR modeling approach, retaining covariates with *p*-values < 0.05. The positive or negative coefficients were estimated. The GWR analysis is a spatial statistic used to identify neighborhoods subjected to higher risk. In our study, increases in open locations associated with increases with specific socioeconomic variables represent higher risk. A positive coefficient indicated that an increased covariate was associated with an increase in open-closed. In contrast, negative coefficients indicated that an increased covariate was associated with a decrease in open-closed.

## Results

**Fig. 2:**
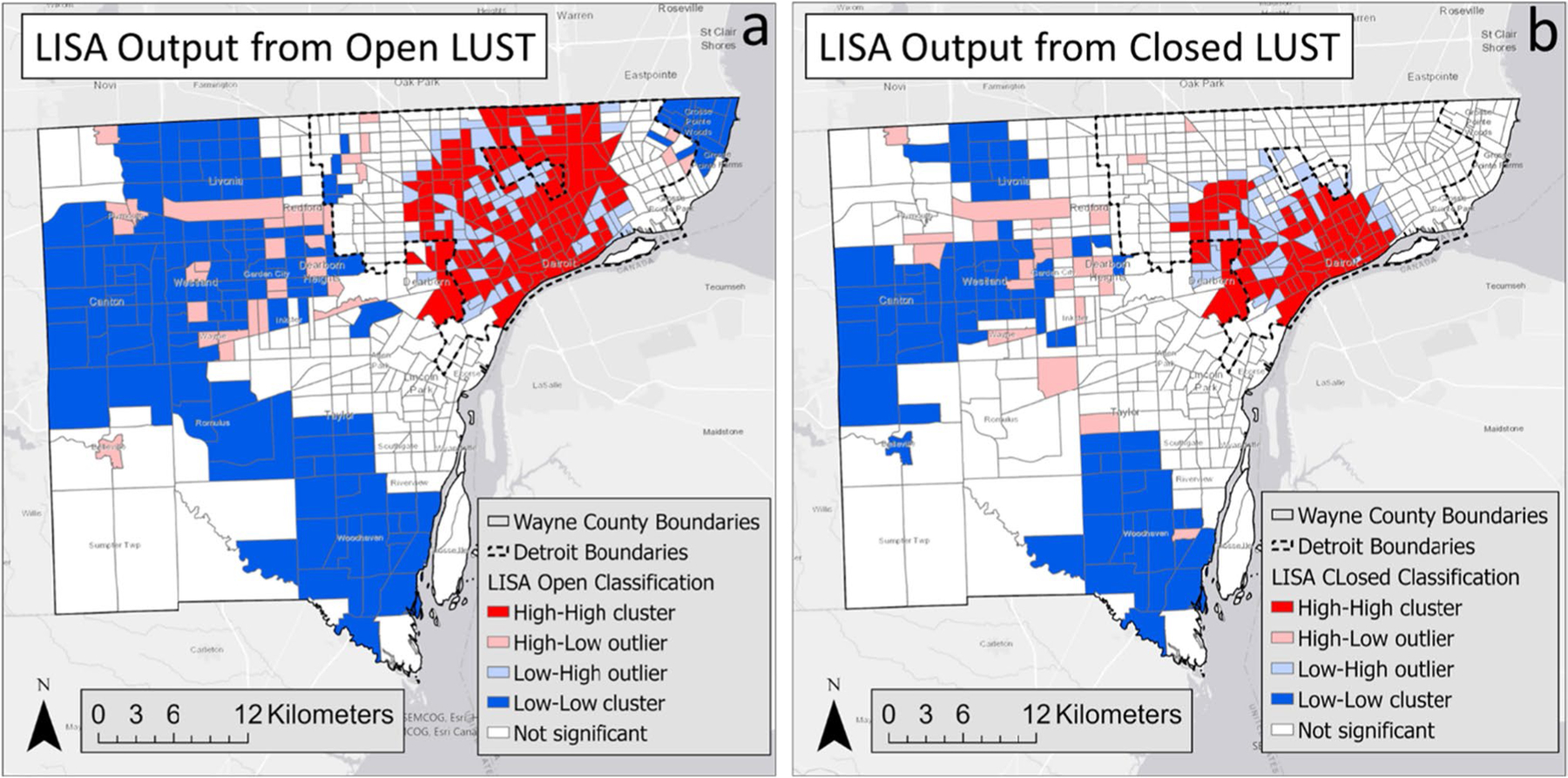
LISA output from the Local Moran’s I analysis and Local Moran’s I for open (**a**) and closed (**b**)

A total of 610 census tract locations, which contained both open and closed sites, were evaluated in this analysis of Wayne County. Both the LUST open and LUST closed datasets identified spatial clusters and outliers in Wayne County using LISA. Clusters are identified with the bold red and blue census tracts, while outliers are identified with the lighter pink and light blue census tracts (Fig. 2).

The Open LUST dataset revealed statistically significant H–H concentrations throughout the central section of the city of Detroit (Fig. 2a). L-L concentrations are primarily located in the northwestern, southern, and the northeast neighborhoods of Wayne County. There were few H–L census tracts in the western suburbs and a few on Detroit’s east side. There were several low–high locations throughout Detroit.

The Closed LUST dataset identified statistically significant H–H clusters in central and downtown Detroit (Fig. 2b). L-L clusters are in the western suburbs and south-central section of Wayne County. H–L outliers are primarily located in west central suburbs, and L–H outliers are primarily located in neighborhoods surrounding the H–H census tracts in Detroit.

Agreement of LISA classifications within each census tract varied between the open and closed datasets, with the open dataset having a higher frequency of statistically significant tracts when compared to the closed dataset (Table 1). The McNemars test statistically confirmed this difference between LISA open and closed sites with a *p* < 0.001. Higher proportions of H–H, H–L, L–H, and L-L were observed in open sites compared to closed sites. In contrast, fewer N.S. (226) were observed in the open sites compared to 371 in the closed sites, indicating a significant pattern difference between open and closed sites.

**Fig. 3:**
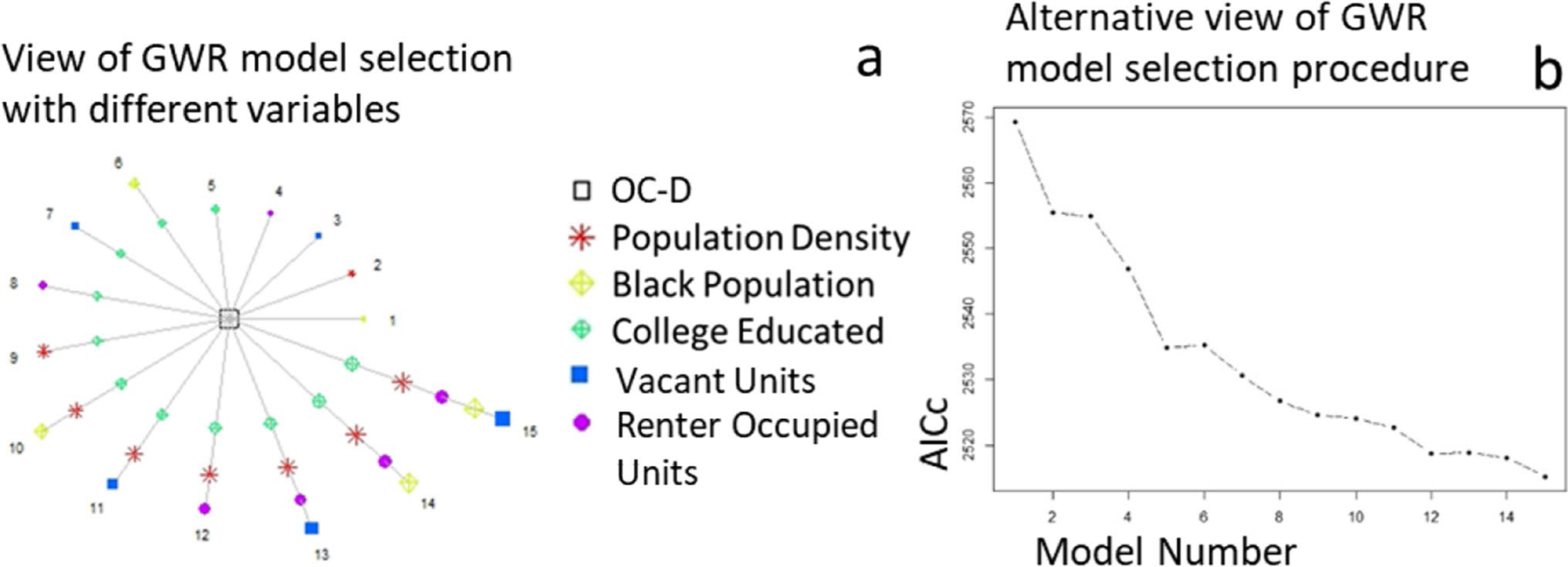
**a** Forward stepwise selection on the left shown by the circle diagram with open-closed difference in the middle and each model iteration with included socioeconomic variables numbered in a circle. **b** An alternative view of this forward stepwise selection is shown on the right with each model number (*x*-axis) and its corresponding AICc (*y*-axis)

High correlations were observed at these sites between the proportion of White and Black residents (*r* = − 0.94), between vacant units and owner-occupied units (*r* = − 0.7), between the proportion of White residents and owner-occupied units (*r* = 0.74), and between the median household value and the college educated population density (*r* = 0.84). Those variables fit the model one at a time along with the other less correlated variables. Minimized AICc was identified with a total of 5 covariates (population density, Black population, college-educated population, vacant unit, and renter unit), resulting in the lowest AICc of 2313 and optimal bandwidth (number of census tracts in the local neighborhood) of 239 (Fig. 3). The multivariate model developed in ESRI ArcGIS showed that all five variables were significantly associated with open and closed site density differences.

**Fig. 4:**
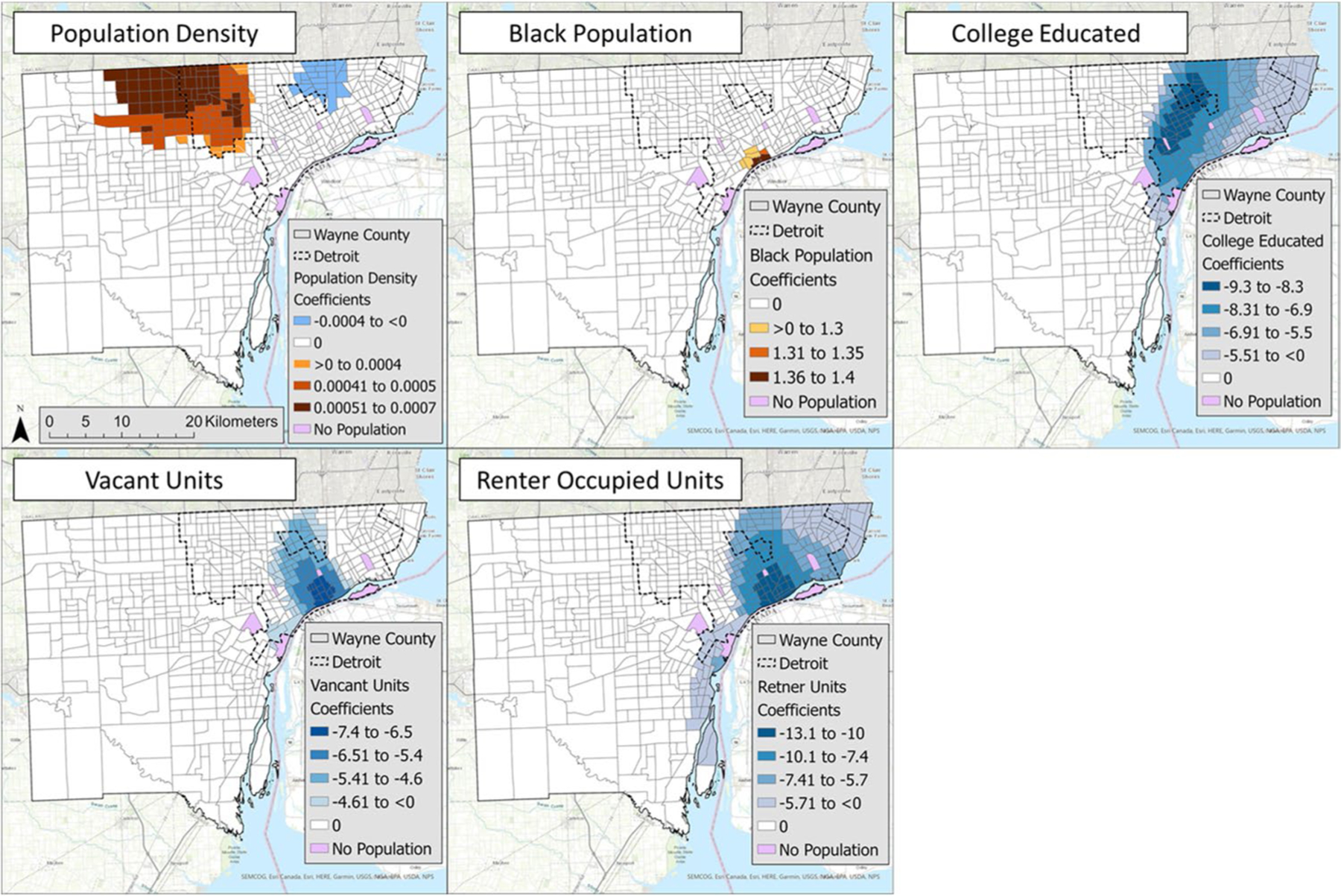
Geographically weighted regression results at the census tract level for all included socioeconomic variables. Orange values indicate a positive correlation, while blue values indicate a negative correlation

The GWR results identified census tracts with significant local factors and the direction of their relationship (Fig. 4). The orange colors indicate a positive association, while blue colors are a negative association, and no color means nonsignificant census tracts. The darker color indicates a stronger relationship, such as a higher/lower coefficient. The results show that population density has positive and negative associations in distinct neighborhoods (Fig. 4). The Black population positively correlates with open-close differences in downtown Detroit. College population, vacant unit, and renter-occupied unit all have a negative association with open-close difference, with the greatest association in central/south-central Detroit.

## Discussion

This analysis aims to provide a geospatial analysis to study the association of environmental remediation with socioeconomic conditions using Wayne County’s LUST spatial data. Previous studies assessed the impact of brownfields on marginalized communities (Lee and Mohai 2011; Lodge et al. 2022; Wilson et al. 2013). Specifically, in Detroit, Lee and Mohai (2013) evaluated socioeconomic dimensions of brownfield cleanup. Our study further expands on evaluating cleanup through spatial modeling and the evaluation of neighborhood scale influences. While closed facilities represent a regulatory endpoint, closed sites often contain residual environmental pollutants creating sustainability and environmental justice concerns (Favara et al. 2019; Smith 2019). Spatial analysis techniques provide environmental justice research preliminary data to identify inequalities in the distribution of environmental hazards (Brender et al. 2011).The techniques outlined in this paper represent example approaches to examine environmental remediation trends spatially at the neighborhood level. The environmental justice research data provides input that describes the distribution of environmental hazards among urban neighborhoods instead of focusing on the contaminant levels of individual properties, which is the typical manner in which current brownfield remediation is undertaken.

## Spatial distribution of LUST

The first goal of this study was to determine if there is a spatial pattern to LUST sites in Wayne County. LISA confirmed clustering and outliers of both open and closed sites. LISA identified distinct differences of high brownfield clustering in the city of Detroit versus low clustering outside of the city. This outcome follows the spatial density of brownfield sites in Wayne County. LISA provides information related to the location of spatial clusters and outliers and the types of spatial correlation. Local statistics are important because the magnitude of the spatial autocorrelation was not necessarily uniform over the study area (e.g., on the county scale) (Anselin 1995; Ord and Getis 1995). LISA identified detailed variations of clustering in the locally defined geography in Wayne County, enabling the assessment of significant local spatial clustering around specific neighborhoods and municipalities.

The second goal of the study was to determine if there is a clustering and outlier category difference in the spatial census tract locations with both open and closed LUST sites. While the general clustering pattern identified by LISA appeared to be similar between both maps, McNemar’s test statistics showed significant discordance in the LISA category between open and closed sites. For example, N.S. was identified in 371 US census tracks. Among those N.S. from closed sites, only 213 NS were identified at the open site, and 50, 17, 26, and 65 were identified as H.H., H.L., L.H., and L.L. categories, respectively. The paired use of LISA clustering and outlier categories with the McNemar’s test statistics demonstrated a useful application to study in-depth features that are similar (clustering) and dissimilar for LISA density (number of sites per surface area of tract). Future studies can apply this approach to additional difference variables (e.g., cancer incidence).

In addition, non-agreement between LISA categories can reveal an important disconnect in the closure or remediation activity in a specific neighborhood. For example, in our study, central Detroit and sections west of the city center identified an H–H clustering for both open and closed sites, indicating a focus on remediation in these neighborhoods. This is in contrast to north-central Detroit with H–H open neighborhood clustering but without a similarly high closure rate.

## Variables impacting LUST

The third goal of the study aimed to evaluate LUST spatial patterns against local socioeconomic and property development factors. Our study hypothesized that closed sites are associated with increased property ownership, wealth, and education outcomes compared to open sites. Negative associations were observed between open-closed and college-educated residents, confirming the hypothesis for this variable (Fig. 4). However, increases in vacant-unit residents and renter-occupied residents were also associated with open-closed. Population density was the only variable with both positive and negative coefficients across geographic regions. The magnitude of the correlations differed with low values for population density and Black residents compared to college educated residents, vacant units, and renter-occupied unit residents.

The GWR models identified two neighborhoods of potential risk, defined by statistically significant locations with elevated open-closed ratios controlling for other socioeconomic variables. The first neighborhood was clustered in north central Wayne County, indicating statistically significant associations between higher population density and increases in open-closed difference (Fig. 4). The second neighborhood, a small cluster of census tracts located along the Detroit River, featured increased Black population with increases in the open-closed difference (Fig. 4). Both neighborhoods show an increase in the socioeconomic variable with increasing open or unresolved brownfield locations relative to the closed sites. Contrary spatial trends were noted in other GWR models where associations among population density, college-educated, and renter-occupied units were significant on the east side of Wayne County. Increased vacant units were related to a closed site toward downtown and central Detroit. Given the large population in the city’s central neighborhoods, this result may be attributed to the under-reporting of brownfields surrounding vacant units in other regions of the city. These results demonstrate the usefulness of GWR in discerning remediation trends associated within local-level neighborhoods in urban centers.

Understanding scale is essential when evaluating the impacts of brownfield sites and developing a community engagement strategy. This paper took a county-wide approach to evaluate brownfield impacts on the census tract scale. GWR provides an effective way to explore spatial variability compared to traditional regressions designed for non-spatial data (Comer and Moran 2017; Gilbert and Chakraborty 2011). Previous work has noted that negative property values were associated with brownfields on a micro-neighborhood scale (0.5 square miles, or 6–9 urban city blocks) (Woo and Lee 2016). However, when cleanup occurred, housing values increased nationally by an average of 5.0 to 11.5% at a highly localized level (Haninger et al. 2017). This paper considered brownfields on a larger scale, and increasing the spatial detail of the analysis may yield stronger correlations in future works.

## Limitations of the dataset

The dataset is limited to LUST locations provided by EGLE’s online GIS database clearing house, which was revised in February 2021. The census data is limited to modeled data from the 2019 ACS based on the 2010 US Census. While this data is several years old, it provides the best estimate of current community demographics for our timeframe of interest.

Another important variable not assessed in this study impacting health is severity of contamination. While there is site specific metadata associated with these LUST sites, the contaminant information is frequently missing. We hope that increased interest in understanding brownfields in neighborhoods will make this type of data accessible in future works.

This paper sought to understand the distribution of neighborhood-scale environmental remediation. Our current research is confined to an urban county in MI, and new local environmental and socioeconomic conditions could influence open and closed status differently. However, we believe that this spatial approach can serve as a national example for informing communities about neighborhood spatial variability of brownfields and factors that may influence remediation.

## Conclusions and future perspectives

The following was accomplished in this review of LUST sites: (1) brownfield distribution was successfully identified, (2) differences in spatial patterns between closed and open sites were quantified, and (3) LUST spatial patterns were associated to local socioeconomic and property development factors. Spatial statistics provided a method for evaluating brownfield remediation in Wayne County. LISA provided an overview of open and closed locations, while the GWR evaluated the relation between socioeconomic differences and brownfield cleanup. The datasets were limited due to access and quality of information at the state level, but the data represent a diverse US-based example of a historic brownfield program.

Remediation priorities are usually given to the most marketable brownfield sites (McCarthy 2009) and brownfield remediation projects, while under the oversight of EGLE, these are still framed as economic improvement projects rather than environmental improvement projects (Lee and Mohai 2012). Examples in MI include the Clean Michigan Initiative and Brownfield Redevelopment Authority under EGLE and the Brownfield Michigan Business Tax Credits Michigan Economic Development Corporation. In addition, the economic potential and external interests often reflect a particular moment that frequently overtakes environmental concerns in prioritization (Adams and Payne 2011). As a result, funding prioritization would be well served to employ this data for prioritizing brownfield remediation actions.

Considering the brownfields’ environmental health hazards, there is a need for public knowledge of environmental health concerns (Bogar et al. 2017; Ratnapradipa et al. 2015). While many public municipalities and governmental organizations have open access data regarding brown-fields, new and alternative platforms for communicating the environmental mapping work are needed (Geekiyanage et al. 2020; Schultz et al. 2018). Examples from MI include EGLE’s Story Map site, released in 2021 (EGLE 2021). The Story Maps integrate text, images, live maps, and additional content into an interactive narrative format designed to reach wider audiences.

This project adopted a large-scale approach to review LUST locations. Future research could focus on specific communities to better understand the interactions between local demographic variables, municipal land use decision-making, and risk factors. Future statistical work, land use analysis, and targeted interview sampling will provide the basis for further understanding the variables impacting the closure rate of brownfield locations in Wayne County.

The results of this study underscore how brownfield remediation approaches can be more inclusive of environmental justice concerns through a nuanced understanding of environmental contaminants at the neighborhood level. Current approaches to brownfield remediation are largely centered on the economic potential of individual properties. The data presented here provide a pathway for environmental justice researchers to understand better the environmental remediation needs within urban communities and beyond individual property lines. Shifting the focus to the neighborhood level is important to address environmental contaminant issues more systemically. This approach allows for a more effective approach to the health hazards imposed in neighborhood communities by the presence of environmental contaminants.

## Figures and Tables

**Table 1:** Agreement table between LISA categories for open sites and closed sites

LISA distribution category						
Closed sites	Open sites					
	H-H	H-L	L-H	L-L	N.S.	Total
H-H	59	0	15	0	3	77
H-L	0	9	0	12	3	24
L-H	24	0	24	0	4	52
L-L	0	6	0	77	3	86
N.S.	50	17	26	65	213	371
Total	133	32	65	154	226	610

## Data Availability

The data used in this publication is publicly available from Michigan’s Department of Environment, Great Lakes, and Energy (link: https://www.michigan.gov/egle/maps-data/environmental-mapper), and the US Census Bureau (link: https://www.census.gov/).
